# Patterns of Circadian Variation in 24-Hour Ambulatory Blood Pressure, Heart Rate, and Sympathetic Tone Correlate with Cardiovascular Disease Risk: A Cluster Analysis

**DOI:** 10.1155/2020/4354759

**Published:** 2020-09-22

**Authors:** Myung Han Hyun, Jun Hyuk Kang, Sunghwan Kim, Jin Oh. Na, Cheol Ung Choi, Jin Won Kim, Eung Ju Kim, Seung-Woon Rha, Chang Gyu Park, Eunmi Lee, Hong Seog Seo

**Affiliations:** ^1^Department of Internal Medicine, Korea University Medical Center, Seoul 152-703, Republic of Korea; ^2^Department of Statistics, Korea University, Seoul 136-705, Republic of Korea; ^3^Division of Cardiology, Department of Internal Medicine, Korea University Guro Hospital, Seoul 152-703, Republic of Korea; ^4^Division of Cardiology, Department of Internal Medicine, Wonkwang University Sanbon Hospital, Gyeonggi-do 435-040, Republic of Korea; ^5^Graduate School of Converging Science and Technology, Korea University–Korea Institute of Science and Technology (KU-KIST), Seoul 136-791, Republic of Korea; ^6^Future Convergence Research Division, Korea Institute of Science and Technology, Seoul 136-791, Republic of Korea

## Abstract

To investigate whether specific time series patterns for blood pressure (BP), heart rate (HR), and sympathetic tone are associated with metabolic factors and the 10-year risk of atherosclerotic cardiovascular disease (ASCVD). A total of 989 patients who underwent simultaneous 24-hour ambulatory BP and Holter electrocardiogram monitoring were enrolled. The patients were categorized into sixteen groups according to their circadian patterns using the consensus clustering analysis method. Metabolic factors, including cholesterol profiles and apolipoprotein, were compared. The 10-year ASCVD risk was estimated based on the Framingham risk model. Overall, 16 significant associations were found between the clinical variables and cluster groups. Age was commonly associated with all clusters in systolic BP (SBP), diastolic BP (DBP), HR, and sympathetic tone. Metabolic indicators, including diabetes, body mass index, total cholesterol, high-density lipoprotein, and apolipoprotein, were associated with the four sympathetic tone clusters. In the crude analysis, the ASCVD risk increased incrementally from clusters 1 to 4 across SBP, DBP, HR, and sympathetic tone. After adjustment for multiple variables, however, only sympathetic tone clusters 3 and 4 showed a significantly high proportion of patients at high risk (≥7.5%) of 10-year ASCVD (odds ratio (OR) = 5.90, 95% confidential interval (CI) = 1.27–27.46, and *P* value = 0.024 and OR = 15.28, 95% CI = 3.59–65.11, and *P* value < 0.001, respectively). Time series patterns of BP, HR, and sympathetic tone can serve as an indicator of aging. Circadian variations in sympathetic tone can provide prognostic information about patient metabolic profiles and indicate future ASCVD risk.

## 1. Introduction

Circadian variations in blood pressure (BP) and heart rate (HR) are associated with aging and metabolic abnormalities such as obesity, insulin resistance, and dyslipidemia [1–3]. In addition, diurnal variations in BP and HR are known to be associated with autonomic dysfunction that might contribute to cardiovascular disease [4–6]. Previous observation trials have shown that an abrupt morning BP surge on waking and a dramatic BP drop at night (10%–20%) correlate with major adverse cardiovascular events [7–9]. Thus, BP, HR, and autonomic dysfunction (mixture of sympathetic and vagal activity) variability could be significantly associated with clinical cardiovascular disease (CVD) risk factors, such as age, sex, body mass index, serum glucose level, serum insulin level, and cholesterol level that reflect metabolic and autonomic abnormalities [10–12].

In theory, one-time measurements of BP and HR in a clinical setting might not provide enough information to accurately diagnose and manage hypertension. On the other hand, 24-hour ambulatory BP (AMBP) recordings with a simultaneous 24-hour Holter electrocardiogram (EKG) can exactly measure the time series patterns of BP, HR, and sympathetic tone variability at home, at work, and while asleep [13]. The conventional approaches to analyzing times series patterns consider only the day-to-night ratio or day-to-night variation [14, 15]. Therefore, previous trials could not examine associations between an individual's circadian patterns of BP, HR, and sympathetic tone and clinical cardiovascular risk factors or estimate CVD risks based on how those patterns changed throughout the day and night. Given the uncertainty left by previous analyses, we used the consensus clustering method, which provides pattern recognition tools with consistent reliability by assessing an entire dataset based on each time point; this method has successfully identified gene expression patterns in microarray data [16–18].

Thus, we used data from consecutive patients who underwent 24-hour AMBP and simultaneous 24-hour Holter EKG and applied a cluster analysis to examine the relationships among cardiovascular risk factors, 10-year estimated CVD risk, and the cluster groups.

## 2. Results

### 2.1. Baseline Demographics

The baseline characteristics of the included population are presented in Table 1. Of the 989 patients, the mean age was 55.5 years, 37.7% of them were male, 9.8% had DM, and 39.0% had hypertension. The mean cholesterol profiles for T-C, HDL-C, LDL-C, and triglycerides were 4.88 ± 1.03, 1.42 ± 0.36, 3.02 ± 0.93, and 3.15 ± 1.95, respectively. The mean 10-year ASCVD risk was 7.6%, of which low risk (<7.5%) patients accounted for 67.0% and high risk (≥7.5%) patients accounted for 33.0%.

### 2.2. Four Patterns of SBP, DBP, HR, and Sympathetic Tone Time Series

**Figure 1:**
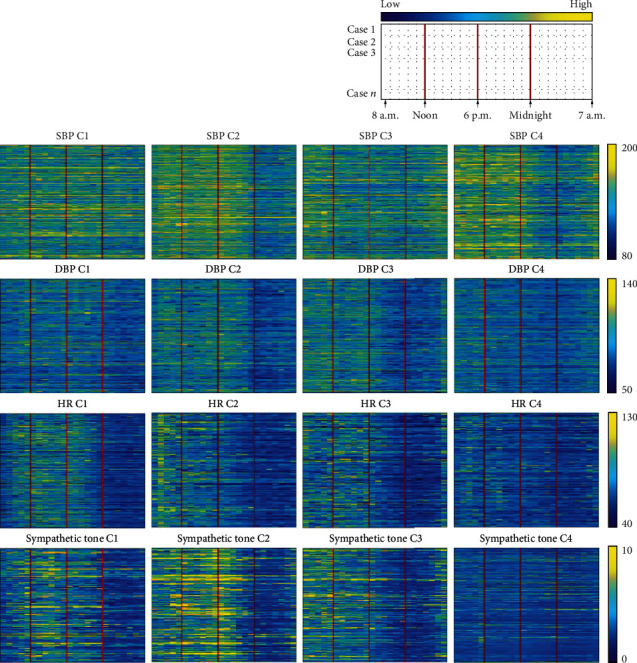
Time series clustering patterns of SBP, DBP, HR, and sympathetic tone. Each time series measurement is represented in matrix form (989 cases [row] × 24 hourly measurements [column]) and visualized using a color map (blue denotes a low value, and yellow denotes a high value for each variable). The measurement time is arranged from left to right in the sequence of morning, afternoon, evening, night, and dawn. Time series patterns are observed to differ by cluster.

**Figure 2:**
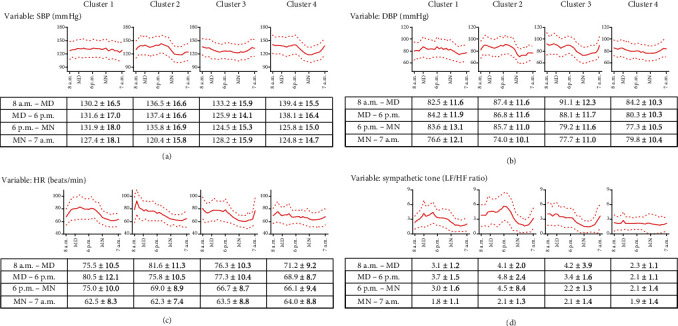
Time series patterns of the SBP, DBP, HR, and sympathetic tone clusters. In each cluster, the time series measurements are visualized as a graph showing mean values (red line) and mean ± 2 standard deviation values (red dotted lines) of the measurements. Data are expressed as mean ± standard deviation. SBP: systolic blood pressure; DBP: diastolic blood pressure; HR: heart rate; LF: low frequency; HF: high frequency; MD: midday; MN: midnight.

The consensus clustering identified four different time series patterns, one each for SBP, DBP, HR, and sympathetic tone (Figure 1). The detailed heat maps of the consensus matrix for each time series pattern are provided in Figure S1–5. The consensus cumulative distribution analysis determined that the number of clusters for each pattern was four (Figure S6). Patients were relatively even distributed among the clusters in the SBP, DBP, and HR groups, whereas for sympathetic tone, more than half of the patients were assigned to cluster 4. The detailed time series patterns by cluster are detailed in Figure 2. In SBP, clusters 1 and 3 showed moderately constant SBP during the 24-hour AMBP analysis. In cluster 2, the patients' MN–7 a.m. BP dropped from 131.9 mmHg to 127.4 mmHg. On the other hand, cluster 4 showed BP drops at both 6 p.m.–MN and MN–7 a.m., from 138.1 mmHg to 125.8 mmHg and 124.8 mmHg, respectively. In the DBP analysis, clusters 1, 2, and 3 have drops in the MN–7 a.m. BP, with clusters 2 and 3 dropping more than cluster 1. The patients in cluster 4 had a relatively constant DBP throughout the 24-hour AMBP test. In the HR analysis, all clusters had an HR increase from MD–6 p.m.. After MN, however, all clusters showed HR drops, though the amount of HR reduction varied by cluster. In the sympathetic tone analysis, clusters 1, 2, and 3 had higher differences between the MD–6 p.m. and MN–6 a.m. values than cluster 4. The highest difference for mean sympathetic tone between MD–6 p.m. and MN–6 a.m. was in cluster 2 (4.8 to 2.1), followed by cluster 1 (3.7 to 1.8), and cluster 3 (3.4 to 2.1).

### 2.3. Associations between Clinical Variables and Each Cluster Group

The detailed associations between selected clinical variables and each set of clusters (SBP, DBP, HR, and sympathetic tone) are described in Table 2. In total, we found 16 significant associations between clinical variables and the cluster groups in our one-way ANOVA tests (for continuous variables) and chi-squared tests (for categorical variables): (1) the SBP clusters were associated with age, smoking, hypertension, and triglyceride levels; (2) the DBP clusters with age, smoking, hypertension, prior history of MI, and BMI; (3) the HR clusters with age, smoking, hypertension, dyslipidemia, and ApoA-I levels; and (4) the sympathetic tone clusters with age, DM, BMI, T-C, HDL-C, and ApoB levels.

To gain more detailed information, we conducted multiple comparison analyses for the continuous clinical variables. From the results, we inferred the following associations: (1) old age with SBP cluster 3, SBP cluster 4, DBP cluster 2, DBP cluster 3, DBP cluster 4, HR cluster 3, HR cluster 4, sympathetic tone cluster 3, and sympathetic tone cluster 4; (2) low BMI with DBP cluster 1, DBP cluster 2, and sympathetic tone cluster 4; (3) high triglycerides with SBP cluster 2 and SBP cluster 4; (4) low ApoA-I levels with HR cluster 3; and (5) low ApoB levels with sympathetic tone cluster 4. Among the categorical clinical variables, the clusters were associated with a higher proportion of the following: (1) smoking with SBP cluster 2, DBP cluster 1, DBP cluster 2, and HR cluster 1; (2) DM with HR cluster 4; (3) hypertension with SBP cluster 2, DBP cluster 2, and HR cluster 2; (3) DM with HR cluster 4; (4) dyslipidemia with HR cluster 4; and (5) prior history of MI with DBP cluster 4.

### 2.4. Associations between 10-Year ASCVD Risk and Each Cluster

The estimated 10-year ASCVD risk by cluster is shown in Table 3. Overall, the ASCVD risk increased incrementally from clusters 1 to 4 across the SBP, DBP, HR, and sympathetic tone patterns. In the crude analysis, SBP clusters 3 and 4 had a significantly higher proportion of patients with high (≥7.5%) 10-year ASCVD risk compared with SBP cluster 1 (odds ratio (OR) = 2.32, 95% confidential interval (CI) = 1.50–3.58, and *P* value < 0.001 and OR = 2.88, 95% CI = 1.85–4.49, and *P* value < 0.001, respectively). Similarly, the DBP, HR, and sympathetic tone clusters 3 and 4 had a significantly higher proportion of patients with risk 10-year ASCVD high compared with cluster 1 (all *P* values < 0.01). After adjustment for multiple variables, however, only sympathetic tone clusters 3 and 4 showed a significantly higher proportion of high-risk 10-year ASCVD patients compared with cluster 1 (OR = 5.90, 95% CI = 1.27–27.46, and *P* value = 0.024 and OR = 15.28, 95% CI = 3.59–65.11, and *P* value < 0.001, respectively).

## 3. Discussion

In our time series clustering analyses, we found three notable associations between the time series patterns and clinical variables: (1) age was associated with all patterns (SBP, DBP, HR, and sympathetic tone); (2) metabolic indicators (DM, BMI, serum T-C level, serum HDL-C level, and plasma ApoB level) were associated with the sympathetic tone pattern; and (3) the sympathetic tone clusters were significantly associated with 10-year ASCVD risk. These results indicate that the time series patterns of BP, HR, and sympathetic tone can provide decipherable information about patient clinical status and future ASCVD risk.

The age-related BP change was well described in the Framingham Heart Study. Those investigators suggested that SBP increases linearly from age 30 through 84 years, whereas DBP increases until age 50 to 60 years and decreases after that [19]. In addition to age-related BP changes, numerous studies have found that a morning BP surge, especially in the first hour after waking, can predict future cardiovascular events and overall short-term mortality [9, 20]. However, previous studies mainly focused on the absolute values of SBP and DBP change as categorical variables, which are not relevant in analyzing time series patterns from 24-hour AMBP data. Distinct from previous studies, our time series clustering analysis implies that both 24-hour AMBP status and the whole system that regulates BP functions differently with age in particular clusters (SBP cluster 3, SBP cluster 4, DBP cluster 2, DBP cluster 3, DBP cluster 4, HR cluster 3, HR cluster 4, sympathetic tone cluster 3, and sympathetic tone cluster 4). Correspondingly, we found that SBP cluster 4 and DBP clusters 3 and 4, which showed high morning BP (8 a.m.–MD compared to MN–7 a.m.), correlate closely with high 10-year ASCVD risk.

Previous experimental and clinical studies have found that the sympathetic nervous system is involved in the regulation of glucose and lipid metabolism, which can play a significant role in the pathophysiology of cardiovascular disease [21, 22]. A chronic increase in sympathetic nerve discharge from the central nervous system resulted in endothelial dysfunction, metabolic function, and other end-organ damage [23]. Numerous mechanisms have been suggested to explain those connections, including the relationship between sympathetic tone activity and fructose metabolism, electrical instability, and the renin-angiotensin aldosterone system [24–26]. Our study shows that the 24-hour pattern of sympathetic tone is associated with five indicators of metabolism: DM, BMI, plasma ApoB level, serum cholesterol level, and serum HDL-C level. In general, human circadian rhythms correlate with sympathetic tone activity, surging during the day and dropping at night. However, our analysis supports the finding that impaired circadian sympathetic tone activity could be linked to a high risk for future CVD; sympathetic tone cluster 4 was characterized by relatively constant sympathetic tone activity throughout the 24-hour monitoring period and carried the highest CVD risk. The fact that these indicators were not associated with the other time series patterns seems to indicate the dominant involvement of sympathetic tone in regulating metabolism. Therefore, our clustering analysis for 24-hour sympathetic tone activity identified a risk group for metabolic abnormality and future ASCVD: clinicians should pay particular attention to patients with low diurnal variation in their sympathetic tone.

Recently, consensus clustering has gained popularity in bioinformatics research for analyzing large datasets, although it has rarely been used in cardiovascular research. In this study, we successfully applied it to analyze clinical data, determining patterns in time series data for SBP, DBP, HR, and sympathetic tone. The patterns we discerned in this way could be used for diagnostic purposes or to determine treatment plans. In our analyses, we identified four clusters based on two technical indices of consensus clustering (consensus CDF and delta area). Although a greater number of clusters could describe more detailed patterns in the time series data, it could also complicate interpretation of the results. Therefore, the number of clusters should be appropriately determined, taking into account the nature of the clinical data and the technical indices.

This study has several limitations. The exact underlying mechanisms for the associations we found remain unexplained. Although previous studies have revealed that a morning surge, midnight drop, and inconsistency in diurnal variation in 24-hour AMBP and 24-hour sympathetic tone correlate with metabolic abnormalities and cardiovascular disease risk, further mechanistic studies are warranted. In addition, ethnic differences should be taken into consideration. Even though our analysis revealed novel associations between time series patterns of BP, HR, and sympathetic tone and clinical variables, we included only the Asian patients from a single tertiary center. Larger scale multicenter trials with variable ethnicity are needed. Nonetheless, this is the largest cohort study to find that cardiovascular disease risk factors and metabolic status correlate with time series patterns of SBP, DBP, HR, and sympathetic tone, and we are the first to use the consensus clustering method with this type of data.

In conclusion, we found that time series patterns of BP, HR, and sympathetic tone are associated with age. Additionally, metabolic indicators correlated with the time series sympathetic tone patterns in ways that could predict future cardiovascular risk. Circadian variations in clinical 24-hour BP, HR, and sympathetic tone data can be analyzed using clustering techniques to identify patients at risk for future cardiovascular disease.

## 4. Materials and Methods

### 4.1. Study Population and Definitions

From January 2011 until December 2013, 3,687 consecutive patients who visited the outpatient department at the Cardiovascular Center at Korea University Guro Hospital, Seoul, Republic of Korea, were enrolled in a prospective observational cohort study. The population that qualified for inclusion in this study were those with available baseline clinical cardiovascular risk factors and 24-hour AMBP and 24-hour Holter monitoring data. Among the 1,081 patients with available simultaneous time series data, ninety-two patients were excluded because they were missing baseline data or had incomplete measurement data. Thus, 989 patients were eligible for this study. The study protocol was approved by the Institutional Review Board at Korea University Guro Hospital (#KUGH15310) and conforms to the ethical guidelines of the Declaration of Helsinki and its later amendments.

The 24-hour AMBP was recorded using a validated oscillometric device (TONOPORT V, Firmware Version 2.0, GE Medical Systems IT, Milwaukee, WI, USA). The device took automatic BP measurements every 30 minutes from 7 a.m. to 10 p.m. (in daytime) and every 60 minutes from 10 p.m. to 7 a.m. (while asleep). A continuous ambulatory electrocardiogram was recorded over the same 24-hour period as the BP measurements were taken using a Seer Light recorder (GE Healthcare, Milwaukee, WI, USA) while the subjects performed their usual daily activities. The recording collected three channels of electrocardiographic data. The HR variability analysis was performed with the MARS analysis program (GE Healthcare). One analyst who was blinded to the clinical histories of the patients reviewed and edited all the data. We used recommendations from the European Society of Cardiology and the North American Society of Pacing Electrophysiology to evaluate sympathetic tone analysis [27, 28]. After removing atrial and ventricular premature complexes, the normal-to-normal (NN) intervals between 150 and 5000 milliseconds with NN ratios between 0.8 and 1.2 were included. Time domain HRV indices, SDNN (standard deviation of all NN intervals; estimate of overall HR variability), SDANN (standard deviation of the averages of NN intervals in all 5-minute segments of the entire recording; estimate of long-term components of HR variability), and RMSSD (square root of the mean of the sum of the squares of differences between adjacent NN intervals; estimate of short-term components of heart rate variability), were calculated from the 24 h Holter ECG recordings. Accordingly, the following domain indices were measured hourly to evaluate sympathetic tone: very low frequency (VLF) of 0.003 to 0.04 Hz, low frequency (LF) of 0.04 to 0.15 Hz, high frequency (HF) of 0.15 to 0.40 Hz, and the LF/HF ratio (LF/HF) in the frequency domain (fast Fourier transform). Time domain analysis was analyzed by the fast Fourier transformation and filtered by the Hamming window [27, 28]. The 10-year CVD risk estimation was calculated according to the Framingham risk score [29]. The Framingham risk model comprises age, sex, smoking status, systolic blood pressure (SBP), total cholesterol (T-C), high-density lipoprotein (HDL-C), and antihypertensive medication to assess the 10-year risk for developing atherosclerotic CVD (ASCVD) [29]. Because this score estimates the 10-year CVD risk for patients aged 40 to 79 years, patients younger than 40 or older than 79 were considered to be 40 and 79, respectively. A 10-year estimated risk equal to or more than 7.5% was defined as high risk, and a risk of less than 7.5% was defined as low risk [29].

### 4.2. Grouping of the Time Series Data

Hourly measured SBP, diastolic blood pressure (DBP), HR, and sympathetic nervous system tone constituted the time series variables for this study (each time series variable thus had 24 values per subject). The time series SBP, DBP, and HR were based on the 24-hour AMBP data, and time series sympathetic tone was obtained by dividing the LF component by the HF component in the Holter data [30].

Using the consensus clustering method, which is widely used to investigate patterns in large datasets, we grouped the 989 patients for each parameter according to their time series patterns. For the consensus clustering, the partitioning around methods clustering algorithm and Pearson distance measurement were adopted, and 1,000 subsamplings were conducted. The optimal number for each group was determined based on the consensus cumulative distribution function (CDF) and delta area. All analyses were performed using the ConsensusClusterPlus package in R [31].

### 4.3. Statistical Analyses

The chi-squared test was used to determine the associations between categorical clinical variables (sex, history of smoking, hypertension, diabetes mellitus (DM), dyslipidemia, prior history of myocardial infarction (MI), and cerebrovascular disease) and each of the four cluster groups (SBP, DBP, HR, and sympathetic tone). The association between continuous clinical variables (age, body mass index (BMI), left ventricular ejection fraction, serum glucose level, plasma insulin level, homeostasis model assessment of insulin resistance, serum T-C level, serum triglyceride level, serum low-density lipoprotein cholesterol (LDL-C) level, serum HDL-C level, plasma apolipoprotein (Apo) A-I level, and plasma ApoB level) and each of the four groups was tested using a one-way analysis of variance (ANOVA) with a subsequent Tukey posthoc test for multiple comparisons. The 10-year ASCVD risk was compared by cluster using a binary logistic regression model adjusted for age, sex, history of smoking, hypertension, DM, dyslipidemia, prior history of MI, cerebrovascular accident, and BMI. Statistical analyses were performed using R version 3.2.2. A *P* value < 0.05 was considered significant, and we show exact *P* values.

## Figures and Tables

**Table 1:** Baseline characteristics of the entire study population.

Variables	Values
Age, year	55.5 ± 13.8
Male, *n* (%)	373 (37.7)
DM, *n* (%)	97 (9.8)
Hypertension, *n* (%)	386 (39.0)
Dyslipidemia, *n* (%)	213 (21.5)
History of MI, *n* (%)	32 (3.2)
CVA, *n* (%)	15 (1.5)
BMI, kg/m^2^	24.1 ± 3.1
History of smoking, *n* (%)	107 (10.8)
LVEF, %	61.6 ± 4.0
Laboratory findings	
FBG, mmol/L	5.71 ± 1.13
Insulin, pmol/L	10.2 ± 5.9
HOMA-IR	2.7 ± 2.0
T-C, mmol/L^a^	4.88 ± 1.03
HDL-C, mmol/L^a^	1.42 ± 0.36
LDL-C, mmol/L^a^	3.02 ± 0.93
Triglyceride, mmol/L^a^	3.15 ± 1.95
ApoA-I, mmol/L^b^	5.05 ± 0.95
ApoB, mmol/L^c^	0.17 ± 0.05
Mean 10-year ASCVD risk, %^†^	7.6
Proportion of low risk (<7.5%) 10-year ASCVD, %^†^	67.0
Proportion of high risk (≥7.5%) 10-year ASCVD, %^†^	33.0

Data are expressed as *n* (%) or mean ± standard deviation. DM: diabetes mellitus; HTN: hypertension; MI: myocardial infarction; BMI: body mass index; CVA: cerebrovascular accident; LVEF: left ventricular ejection fraction; FBG: fasting blood glucose; Insulin: fasting blood insulin; HOMA-IR: homeostasis model assessment of insulin resistance; T-C: total cholesterol; HDL-C: high-density lipoprotein cholesterol; LDL-C: low-density lipoprotein cholesterol; Apo: apolipoprotein; ASCVD: atherosclerotic cardiovascular disease. ^a^To convert to milligram per deciliter, divide by 0.0259. ^b^To convert to milligram per deciliter, divide by 0.0357. ^c^To convert to milligram per deciliter, divide by 0.0019. ^†^According to the 2013 American College of Cardiology/American Heart Association, the cardiovascular risk score calculates the risk of 10-year atherosclerotic cardiovascular disease.

**Table 2:** Characteristics between the significantly associated clinical variables and cluster groups for SBP, DBP, HR, and sympathetic tone.

Variables	SBP clusters				*P* value^∗^	DBP clusters				*P* value^∗^

	C1 (*n* = 213)	C2 (*n* = 301)	C3 (*n* = 250)	C4 (*n* = 225)		C1 (*n* = 210)	C2 (*n* = 232)	C3 (*n* = 225)	C4 (*n* = 322)	
Age, year^†^	50.7 ± 15.3	52.1 ± 13.6	59.8 ± 12.5	60.0 ± 11.0	<0.001^b,c,d,e^	50.8 ± 15.0	51.8 ± 14.1	58.0 ± 11.6	59.6 ± 12.5	<0.001^b,c,d,e^
Male, *n* (%)	74 (34.7)	129 (42.9)	81 (32.4)	89 (39.6)	0.057	76 (36.2)	88 (37.9)	94 (41.8)	115 (35.7)	0.505
History of smoking, *n* (%)^†^	21 (9.9)	51 (16.9)	17 (6.8)	18 (8.0)	<0.001^d,e^	28 (13.3)	35 (15.1)	19 (8.4)	25 (7.8)	0.016^b,e^
DM, *n* (%)	15 (7.0)	27 (9.0)	28 (11.2)	27 (12.0)	0.194	19 (9.0)	15 (6.5)	24 (10.7)	39 (12.1)	0.211
Hypertension, *n* (%)^†^	70 (32.9)	99 (32.9)	113 (45.2)	104 (46.2)	<0.001^b,c,d,e^	68 (32.4)	77 (33.2)	90 (40.0)	151 (46.9)	0.002^c,e^
Dyslipidemia, *n* (%)	36 (16.9)	61 (20.3)	59 (23.6)	57 (25.3)	0.136	34 (16.2)	50 (21.6)	47 (20.9)	82 (25.5)	0.088
Prior history of MI, *n* (%)^†^	7 (3.3)	6 (2.0)	11 (4.4)	8 (3.6)	0.450	2 (1.0)	4 (1.7)	6 (2.7)	20 (6.2)	0.002^c^
CVA, *n* (%)	6 (2.8)	1 (0.3)	5 (2.0)	3 (1.3)	0.127	6 (2.9)	0 (0.0)	2 (0.9)	7 (2.2)	0.054
BMI, kg/m^2†^	23.8 ± 3.3	24.0 ± 3.0	24.0 ± 3.2	24.5 ± 3.0	0.129	23.9 ± 3.2	23.7 ± 3.1	24.7 ± 3.1	24.1 ± 3.1	0.004^b,d^
T-C, mmol/L^a^	4.90 ± 1.06	4.96 ± 1.06	4.79 ± 0.96	4.88 ± 1.03	0.329	4.83 ± 1.00	4.96 ± 1.10	4.90 ± 0.95	4.86 ± 1.04	0.578
HDL-C, mmol/L^a^	1.39 ± 0.35	1.41 ± 0.34	1.45 ± 0.40	1.43 ± 0.34	0.322	1.42 ± 0.37	1.44 ± 0.35	1.40 ± 0.33	1.42 ± 0.37	0.674
LDL-C, mmol/L^a^	3.06 ± 0.95	3.11 ± 0.92	2.92 ± 0.88	2.99 ± 0.95	0.225	2.97 ± 0.87	3.07 ± 0.98	3.03 ± 0.90	3.00 ± 0.95	0.694
Triglyceride, mmol/L^†a^	2.93 ± 1.76	3.49 ± 2.36	2.99 ± 1.80	3.08 ± 1.62	0.007^a,d^	3.06 ± 2.30	3.21 ± 1.88	3.26 ± 1.93	3.09 ± 1.76	0.685
ApoA-I, mmol/L^b^	4.91 ± 0.90	5.06 ± 0.89	5.12 ± 1.01	5.08 ± 0.98	0.167	5.02 ± 0.98	5.11 ± 1.02	4.99 ± 0.90	5.06 ± 0.91	0.629
ApoB, mmol/L^c^	0.17 ± 0.05	0.18 ± 0.05	0.17 ± 0.05	0.17 ± 0.06	0.180	0.17 ± 0.05	0.18 ± 0.05	0.18 ± 0.05	0.17 ± 0.05	0.137

Variables	HR clusters				*P* value^∗^	Sympathetic tone clusters				*P* value^∗^

	C1 (*n* = 348)	C2 (*n* = 202)	C3 (*n* = 173)	C4 (*n* = 266)		C1 (*n* = 112)	C2 (*n* = 171)	C3 (*n* = 193)	C4 (*n* = 513)	
Age, year^†^	51.3 ± 14.5	55.3 ± 13.6	58.6 ± 12.5	59.2 ± 12.1	<0.001^a,b,c,e^	50.5 ± 13.8	51.1 ± 12.8	55.9 ± 11.9	58.0 ± 14.2	<0.001^b,c,d,e^
Male, *n* (%)	143 (41.1)	69 (34.2)	56 (32.4)	105 (39.5)	0.156	45 (40.2)	68 (39.8)	69 (35.8)	191 (37.2)	0.810
History of smoking, *n* (%)^†^	48 (13.8)	16 (7.9)	12 (6.9)	31 (11.7)	0.016^b^	8 (7.1)	20 (11.7)	23 (11.9)	56 (10.9)	0.558
DM, *n* (%)^†^	27 (7.8)	21 (10.4)	13 (7.5)	36 (13.5)	0.067	7 (6.3)	7 (4.1)	14 (7.3)	69 (13.5)	0.001^e^
Hypertension, *n* (%)^†^	113 (32.5)	76 (37.6)	64 (37.0)	133 (50.0)	<0.001^c,f^	35 (31.3)	57 (33.3)	83 (43.0)	211 (41.1)	0.120
Dyslipidemia, *n* (%)^†^	60 (17.2)	41 (20.3)	40 (23.1)	72 (27.1)	0.029^c^	19 (17.0)	34 (19.9)	42 (21.8)	118 (23.0)	0.509
Prior history of MI, *n* (%)	7 (2.0)	6 (3.0)	5 (2.9)	14 (5.3)	0.153	4 (3.6)	5 (2.9)	8 (4.1)	15 (2.9)	0.858
CVA, *n* (%)	3 (0.9)	1 (0.5)	4 (2.3)	7 (2.6)	0.147	1 (0.9)	2 (1.2)	2 (1.0)	10 (1.9)	0.715
BMI, kg/m^2†^	23.9 ± 3.2	24.2 ± 3.2	24.0 ± 2.9	24.3 ± 3.1	0.521	24.7 ± 3.2	24.2 ± 3.0	24.3 ± 3.3	23.8 ± 3.1	0.009^c^
T-C, mmol/L^†a^	4.91 ± 1.07	4.82 ± 0.92	5.03 ± 0.95	4.80 ± 1.08	0.140	5.02 ± 1.24	5.01 ± 1.02	4.96 ± 0.98	4.79 ± 0.99	0.026
HDL-C, mmol/L^†a^	1.44 ± 0.38	1.40 ± 0.33	1.46 ± 0.36	1.38 ± 0.35	0.122	1.46 ± 0.38	1.46 ± 0.39	1.45 ± 0.35	1.39 ± 0.35	0.049
LDL-C, mmol/L^a^	3.03 ± 0.98	2.98 ± 0.85	3.10 ± 0.90	2.97 ± 0.92	0.509	3.08 ± 1.04	3.11 ± 0.95	3.09 ± 0.91	2.94 ± 0.89	0.120
Triglyceride, mmol/L^a^	3.13 ± 1.80	3.19 ± 1.99	3.18 ± 1.81	3.13 ± 2.20	0.980	3.35 ± 2.68	3.04 ± 1.77	3.05 ± 1.78	3.18 ± 1.87	0.561
ApoA-I, mmol/L^†b^	5.08 ± 1.04	4.99 ± 0.86	5.24 ± 0.99	4.90 ± 0.83	0.006^f^	5.10 ± 0.97	5.06 ± 1.07	5.06 ± 0.85	5.03 ± 0.94	0.909
ApoB, mmol/L^†c^	0.17 ± 0.05	0.17 ± 0.05	0.18 ± 0.05	0.17 ± 0.05	0.636	0.18 ± 0.06	0.18 ± 0.06	0.18 ± 0.05	0.17 ± 0.05	0.001^e,f^

Data are expressed as *n* (%) or mean ± standard deviation. ^∗^*P* values are from one-way ANOVA tests for continuous variables and chi-squared tests for categorical variables. ^†^To denote the results of the Tukey posthoc test for continuous variables and Bonferroni posthoc test for categorical variables, values that are significantly different (*P* < 0.05) in a row are marked with different superscript letters: ^a^significant differences between C1 and C2; ^b^significant differences between C1 and C3; ^c^significant differences between C1 and C4; ^d^significant differences between C2 and C3; ^e^significant differences between C2 and C4; and ^f^significant differences between C3 and C4. ^a^To convert to milligram per deciliter, divide by 0.0259. ^b^To convert to milligram per deciliter, divide by 0.0357. ^c^To convert to milligram per deciliter, divide by 0.0019. SBP: systolic blood pressure; DBP: diastolic blood pressure; HR: heart rate; DM: diabetes mellitus; MI: myocardial infarction; CVA: cerebrovascular accident; BMI: body mass index; T-C: total cholesterol; HDL-C: high-density lipoprotein cholesterol; LDL-C: low-density lipoprotein cholesterol; Apo: apolipoprotein.

**Table 3:** Estimated 10-year ASCVD risk according to the SBP, DBP, HR, and sympathetic tone clustering analyses.

	Mean 10-year ASCVD risk score^∗^	Proportion of low risk (<7.5%) 10-year ASCVD^∗^	Proportion of high risk (≥7.5%) 10-year ASCVD^∗^	*P* value	Crude OR for high risk (≥7.5%) 10-year ASCVD^∗^	*P* value	Adjusted OR for high risk (≥7.5%) 10-year ASCVD^∗^^†^	*P* value
SBP clusters								
SBP C1	5.9%	77.4%	22.6%	<0.001	1 (reference)	N/A	1 (reference)	N/A
SBP C2	5.9%	75.6%	24.4%		1.11 (0.71-1.73)	0.653	0.88 (0.30-2.63)	0.881
SBP C3	9.2%	59.6%	40.4%		2.32 (1.50-3.58)	<0.001	0.77 (0.26-2.26)	0.769
SBP C4	9.5%	54.3%	45.7%		2.88 (1.85-4.49)	<0.001	0.88 (0.30-2.60)	0.875
DBP clusters								
DBP C1	5.2%	81.1%	18.9%	<0.001	1 (reference)	N/A	1 (reference)	N/A
DBP C2	6.0%	75.5%	24.5%		1.39 (0.85-2.27)	0.186	1.26 (0.40-4.00)	0.696
DBP C3	8.0%	61.4%	38.6%		2.70 (1.69-4.32)	<0.001	0.98 (0.33-2.93)	0.967
DBP C4	9.9%	55.9%	44.1%		3.38 (2.19-5.21)	<0.001	1.84 (0.66-5.20)	0.246
HR clusters								
HR C1	5.6%	76.5%	23.5%	<0.001	1 (reference)	N/A	1 (reference)	N/A
HR C2	7.5%	68.7%	31.3%		1.49 (0.98-2.24)	0.059	1.59 (0.58-4.38)	0.370
HR C3	8.3%	63.6%	36.4%		1.87 (1.22-2.84)	0.004	1.61 (0.58-4.47)	0.358
HR C4	9.8%	55.0%	45.0%		2.67 (1.84-3.87)	<0.001	2.09 (0.85-5.12)	0.108
Sympathetic tone clusters								
Sympathetic tone C1	4.3%	88.8%	11.2%	<0.001	1 (reference)	N/A	1 (reference)	N/A
Sympathetic tone C2	4.7%	80.6%	19.4%		1.91 (0.90-4.05)	0.091	4.56 (0.88-23.55)	0.070
Sympathetic tone C3	6.8%	70.7%	29.3%		3.28 (1.61-6.68)	0.001	5.90 (1.27-27.46)	0.024
Sympathetic tone C4	9.5%	56.7%	43.3%		6.03 (3.14-11.60)	<0.001	15.28 (3.59-65.11)	<0.001

SBP: systolic blood pressure; DBP: diastolic blood pressure; HR: heart rate; ASCVD: atherosclerotic cardiovascular disease; N/A: not available. ^∗^According to the 2013 American College of Cardiology/American Heart Association, the cardiovascular risk score calculates the 10-year risk of atherosclerotic cardiovascular disease. ^†^Binary logistic regression for adjusted variables: age, sex, history of smoking, hypertension, diabetes mellitus, dyslipidemia, prior history of myocardial infarction, cerebrovascular accident, and body mass index.

## Supplementary Materials

Supplementary Materials Figure S1: consensus color legend. The consensus matrices have rows and columns. The consensus values range from 0 (never clustered together) to 1 (always clustered together) and are represented by colors from white to dark blue in the order of increasing values. Figure S2: heat maps of the consensus matrices for SBP. The consensus matrices for *k* = 2, 3, 4, and 5 are colored according to their values as depicted in Figure S1. The matrices are arranged in the order of the consensus clustering shown atop the heat map. Cluster memberships are marked by colored rectangles between the dendrogram and heat map, as explained in the corresponding legends. Figure S3: heat maps of the consensus matrices for DBP. The consensus matrices for *k* = 2, 3, 4, and 5 are colored according to their values as depicted in Figure S1. The matrices are arranged in the order of the consensus clustering shown atop the heat map. Cluster memberships are marked by colored rectangles between the dendrogram and heat map, as explained in the corresponding legends. Figure S4: heat maps of the consensus matrices for HR. The consensus matrices for *k* = 2, 3, 4, and 5 are colored according to their values as depicted in Figure S1. The matrices are arranged in the order of the consensus clustering shown atop the heat map. Cluster memberships are marked by colored rectangles between the dendrogram and heat map, as explained in the corresponding legends. Figure S5: heat maps of the consensus matrices for sympathetic tone. The consensus matrices for *k* = 2, 3, 4, and 5 are colored according to their values as depicted in Figure S1. The matrices are arranged in the order of the consensus clustering shown atop the heat map. Cluster memberships are marked by colored rectangles between the dendrogram and heat map, as explained in the corresponding legends. Figure S6: consensus cumulative distribution function (CDF) plots and delta area plots for clustering SBP (leftmost column), DBP (middle-left column), HR (middle-right column), and sympathetic tone (rightmost column) data. (A) Each plot shows the CDFs of the consensus matrix for each *k* (indicated by colors), estimated with a histogram of 100 bins. (B) Each delta area plot shows the relative changes in the area under the CDF curve between *k* and *k*-1. Because *k* = 2 has no *k*-1, the total area under the curve rather than the relative increase is plotted.
