# Supplementary material for: Patterns of Circadian Variation in 24-Hour Ambulatory Blood Pressure, Heart Rate, and Sympathetic Tone Correlate with Cardiovascular Disease Risk: A Cluster Analysis
Source: Cardiovasc Ther. 2020 Sep 22;2020:4354759. doi: 10.1155/2020/4354759 (PMC7528127; doi:10.1155/2020/4354759)
Supplement: Supplementary Materials — Figure S1: consensus color legend. The consensus matrices have rows and columns. The consensus values range from 0 (never clustered together) to 1 (always clustered together) and are represented by colors from white to dark blue in the order of increasing values. Figure S2: heat maps of the consensus matrices for SBP. The consensus matrices for k = 2, 3, 4, and 5 are colored according to their values as depicted in Figure S1. The matrices are arranged in the order of the consensus clustering shown atop the heat map. Cluster memberships are marked by colored rectangles between the dendrogram and heat map, as explained in the corresponding legends. Figure S3: heat maps of the consensus matrices for DBP. The consensus matrices for k = 2, 3, 4, and 5 are colored according to their values as depicted in Figure S1. The matrices are arranged in the order of the consensus clustering shown atop the heat map. Cluster memberships are marked by colored rectangles between the dendrogram and heat map, as explained in the corresponding legends. Figure S4: heat maps of the consensus matrices for HR. The consensus matrices for k = 2, 3, 4, and 5 are colored according to their values as depicted in Figure S1. The matrices are arranged in the order of the consensus clustering shown atop the heat map. Cluster memberships are marked by colored rectangles between the dendrogram and heat map, as explained in the corresponding legends. Figure S5: heat maps of the consensus matrices for sympathetic tone. The consensus matrices for k = 2, 3, 4, and 5 are colored according to their values as depicted in Figure S1. The matrices are arranged in the order of the consensus clustering shown atop the heat map. Cluster memberships are marked by colored rectangles between the dendrogram and heat map, as explained in the corresponding legends. Figure S6: consensus cumulative distribution function (CDF) plots and delta area plots for clustering SBP (leftmost column), DBP (middle-left column), HR (middl [file 4354759.f1.docx]

**Supplemental Data for**

**Patterns of Circadian Variation in 24-hour Ambulatory Blood Pressure, Heart Rate, and Sympathetic Tone Correlate with Cardiovascular Disease Risk: A Cluster Analysis**

**Running title:** Patterns of BP, HR, sympathetic tone, and CVD risk

**Authors:** Jun Hyuk Kang, M.D., Ph.D.^1*^; Myung Han Hyun, M.D.^1*^; Sunghwan Kim, Ph.D.^2^; Jin Oh Na, M.D., Ph.D.^3^; Cheol Ung Choi, M.D., Ph.D.^3^; Jin Won Kim, M.D., Ph.D.^3^; Eung Ju Kim, M.D., Ph.D.^3^; Seung-Woon Rha, M.D., Ph.D.^3^; Chang Gyu Park, M.D., Ph.D.^3^; Eunmi Lee, M.D., Ph.D.^4†^; and Hong Seog Seo, M.D., Ph.D.^3, 5, 6†^

**Affiliations:**

^1^Department of Internal Medicine, Korea University Medical Center, Seoul 152-703, Republic of Korea

^2^Department of Statistics, Korea University, Seoul 136-705, Republic of Korea

^3^Division of Cardiology, Department of Internal Medicine, Korea University Guro Hospital, Seoul 152-703, Republic of Korea

^4^Division of Cardiology, Department of Internal Medicine, Wonkwang University Sanbon Hospital, Gyeonggi-do 435-040, Republic of Korea

^5^Graduate School of Converging Science and Technology, Korea University–Korea Institute of Science and Technology (KU-KIST), Seoul 136-791, Republic of Korea

^6^Future Convergence Research Division, Korea Institute of Science and Technology, Seoul 136-791, Republic of Korea

^*^These authors contributed equally to this work.

^†^These authors contributed equally to this work.

**Supplemental Figures**


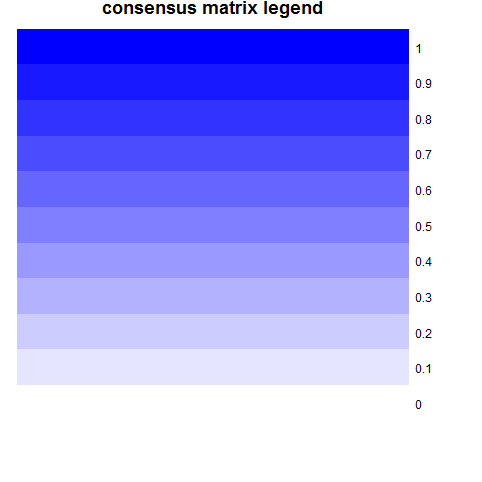


**Figure S1**. Consensus color legend. The consensus matrices have rows and columns. The consensus values range from 0 (never clustered together) to 1 (always clustered together) and are represented by colors from white to dark blue in continuum in the order of increasing values.


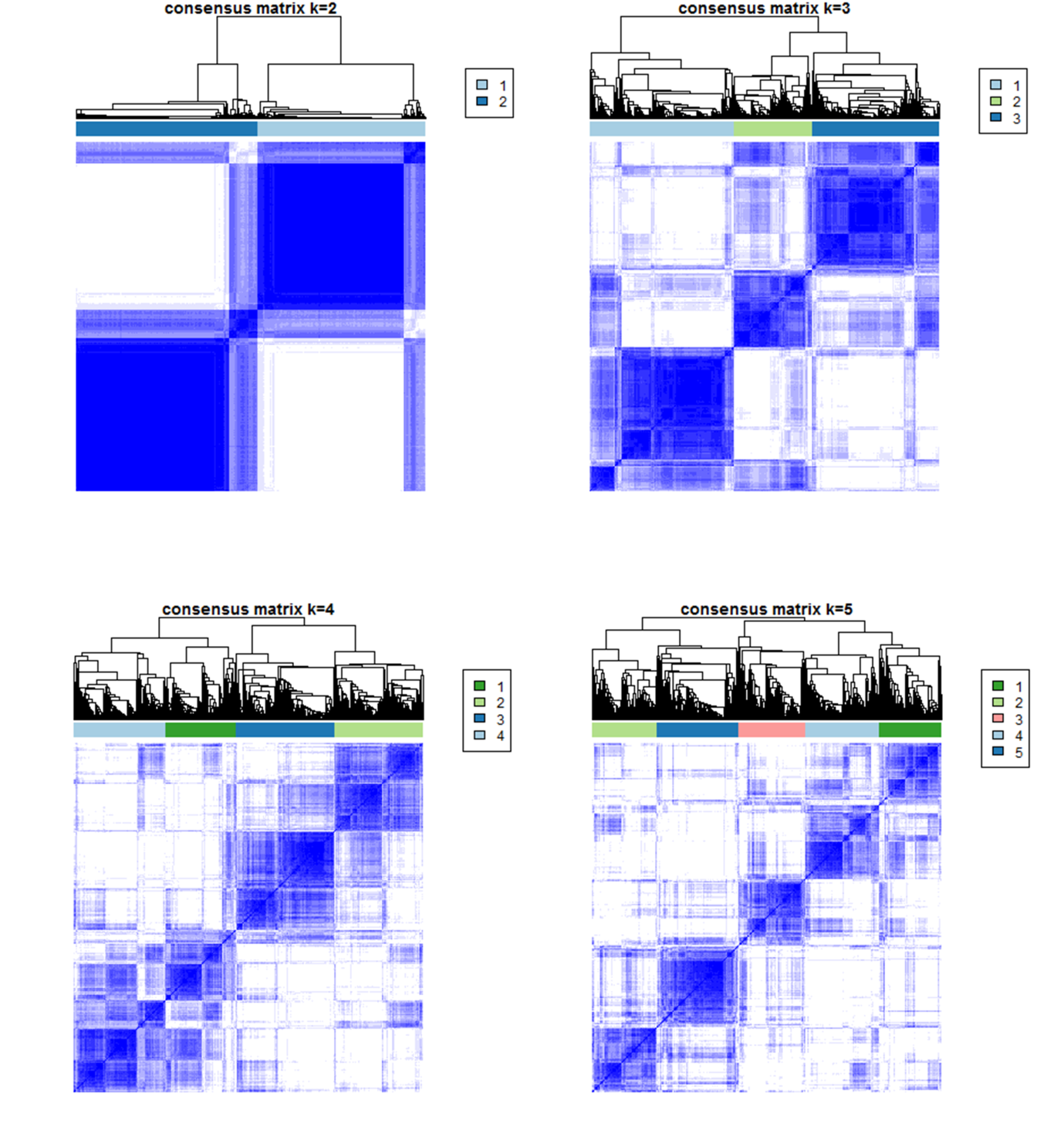


**Figure S2**. Heatmaps of the consensus matrices for SBP. The consensus matrices for k = 2, 3, 4, and 5 are colored according to their values as depicted in Fig. S1. The matrices are arranged in the order of the consensus clustering shown atop the heatmap. The cluster memberships are marked by the color rectangles between the dendrogram and heatmap, of which the meaning of the colors are explained in the corresponding legends placed on the right of the rectangles.


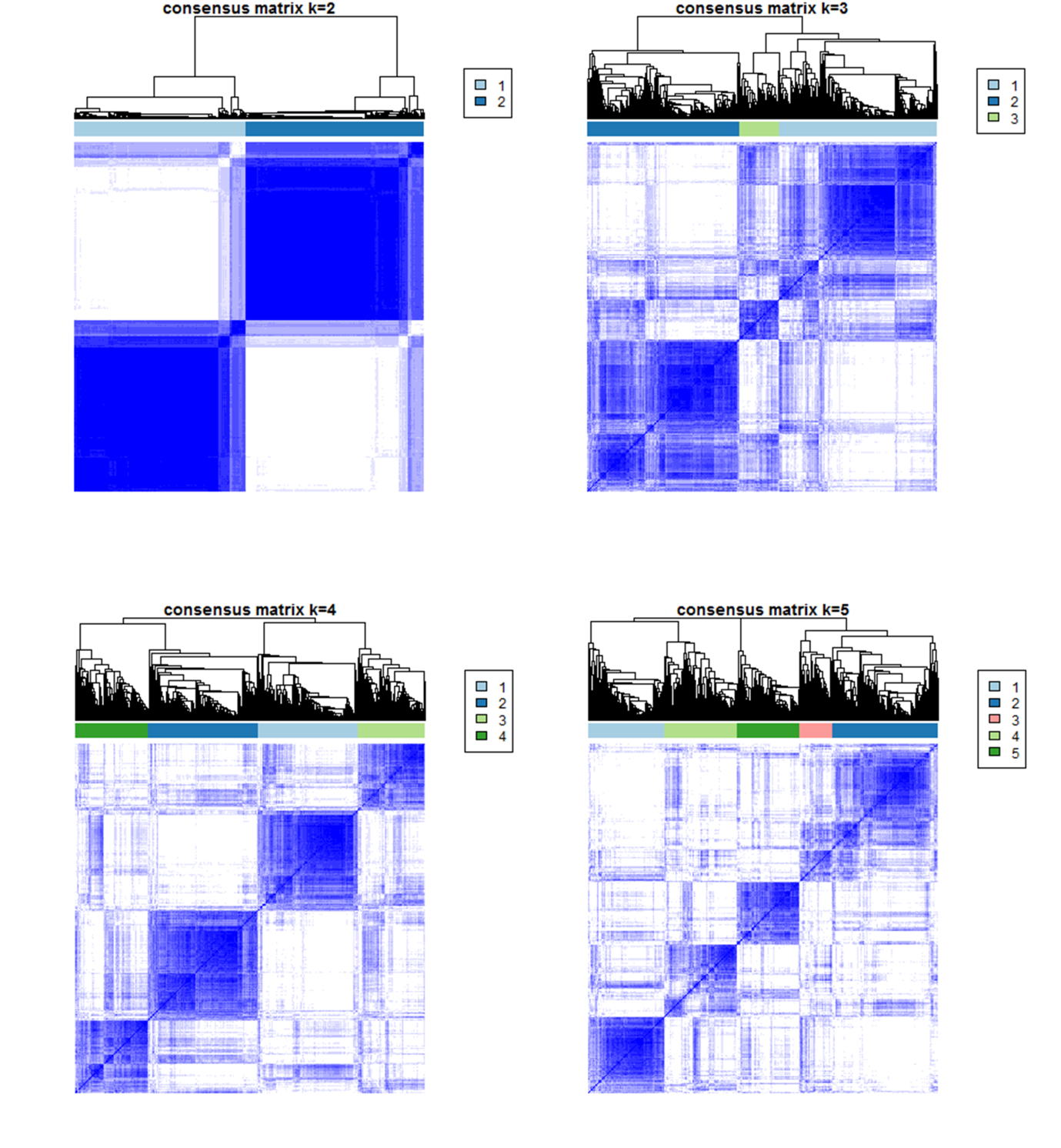


**Figure S3**. Heatmaps of the consensus matrices for DBP. The consensus matrices for k = 2, 3, 4, and 5 are colored according to their values as depicted in Fig. S1. The matrices are arranged in the order of the consensus clustering shown atop the heatmap. The cluster memberships are marked by the color rectangles between the dendrogram and heatmap, of which the meaning of the colors are explained in the corresponding legends placed on the right of the rectangles.


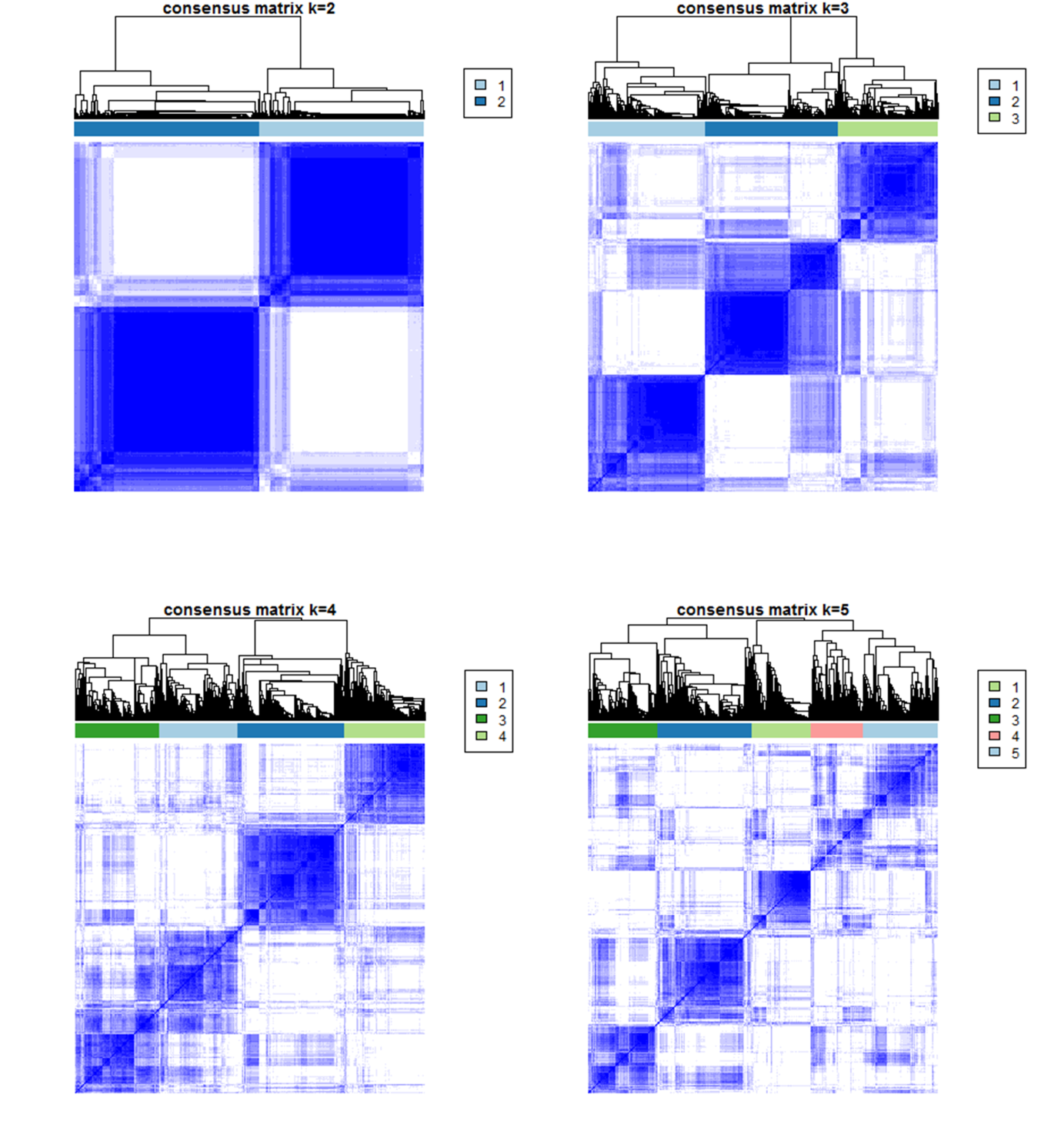


**Figure S4**. Heatmaps of the consensus matrices for HR. The consensus matrices for k = 2, 3, 4, and 5 are colored according to their values as depicted in Fig. S1. The matrices are arranged in the order of the consensus clustering shown atop the heatmap. The cluster memberships are marked by the color rectangles between the dendrogram and heatmap, of which the meaning of the colors are explained in the corresponding legends placed on the right of the rectangles.


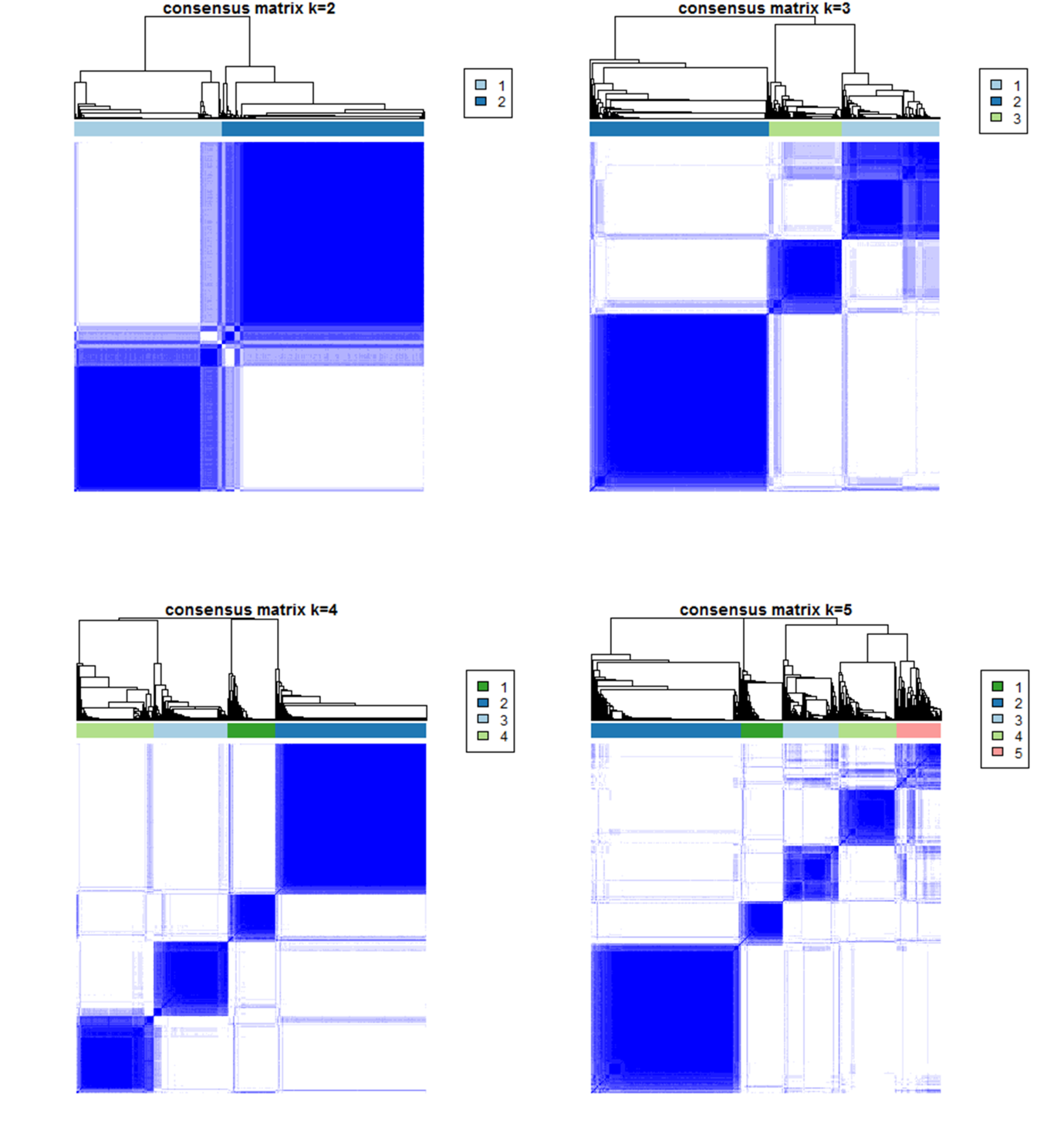


**Figure S5**. Heatmaps of the consensus matrices for sympathetic tone. The consensus matrices for k = 2, 3, 4, and 5 are colored according to their values as depicted in Fig. S1. The matrices are arranged in the order of the consensus clustering shown atop the heatmap. The cluster memberships are marked by the color rectangles between the dendrogram and heatmap, of which the meaning of the colors are explained in the corresponding legends placed on the right of the rectangles.


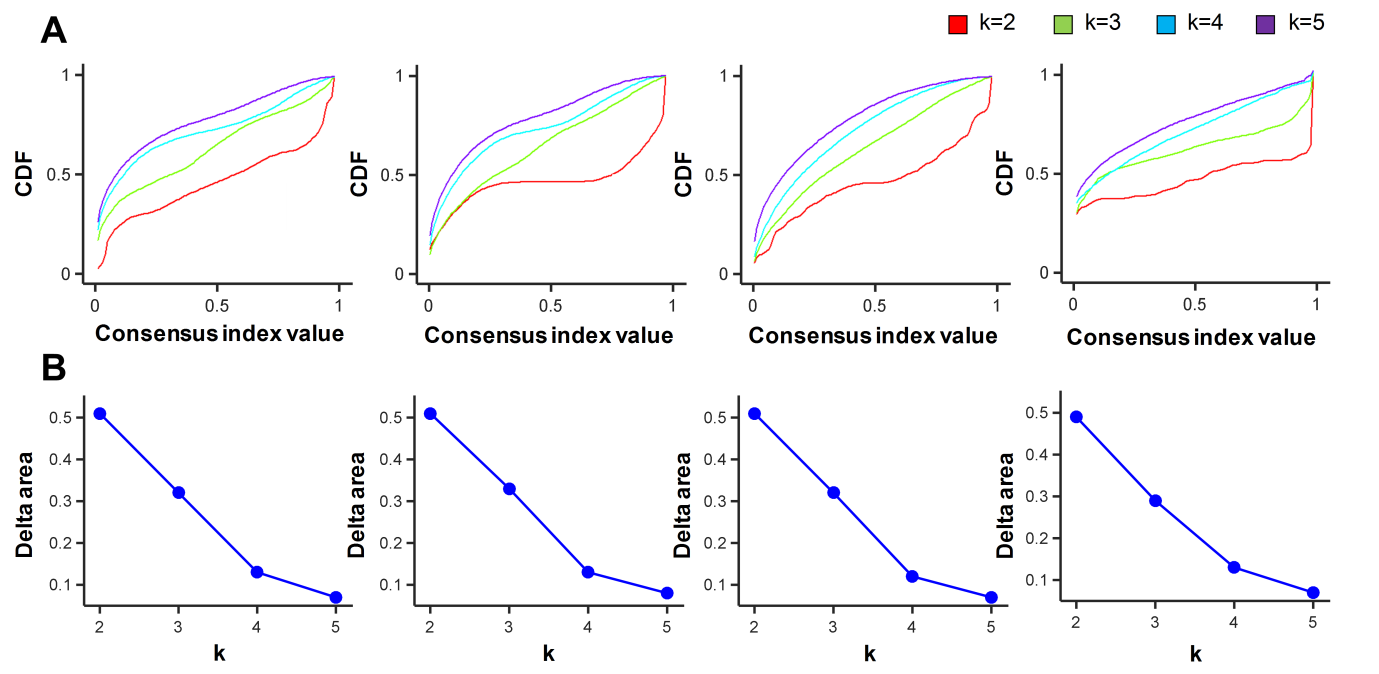


**Figure S6**. Consensus cumulative distribution (CDF) plots and delta area plots. for clustering of SBP (leftmost column), DBP (middle-left column), HR (middle-right column), and sympathetic tone (rightmost column). (A) Each consensus cumulative distribution function (CDF) plot shows the cumulative distribution functions of the consensus matrix for each k (indicated by colors), estimated by a histogram of 100 bins. (B) Each delta area plot shows the relative changes in the area under the CDF curve in comparison between k and k-1. Since there is no k-1 for k=2, the total area under the curve rather than the relative increase is plotted.
